# Unraveling the Relationship Between Teacher-Assigned Grades, Student Personality, and Standardized Test Scores

**DOI:** 10.3389/fpsyg.2021.627440

**Published:** 2021-03-19

**Authors:** Andrea Westphal, Miriam Vock, Julia Kretschmann

**Affiliations:** Department of Education, University of Potsdam, Potsdam, Germany

**Keywords:** Big Five, student personality, teacher-assigned grades, grading practice, conscientiousness, mathematics, German, secondary school

## Abstract

The Big Five personality traits play a major role in student achievement. As such, there is consistent evidence that students that are more conscientious receive better teacher-assigned grades in secondary school. However, research often does not support the claim that students that are more conscientious similarly achieve higher scores in domain-specific standardized achievement tests. Based on the Invest-and-Accrue Model, we argue that conscientiousness explains to some extent why certain students receive better grades despite similar academic accomplishments (i.e., achieving similar scores in domain-specific standardized achievement tests). Therefore, the present study examines to what extent the relationship between student personality and teacher-assigned grades consists of direct as opposed to indirect associations (via subject-specific standardized test scores). We used a representative sample of 14,710 ninth-grade students to estimate these direct and indirect pathways in mathematics and German. Structural equation models showed that test scores explained between 8 and 11% of the variance in teacher-assigned grades in mathematics and German. The Big Five personality traits in students additionally explained between 8 and 10% of the variance in grades. Finally, the personality-grade relationship consisted of direct (0.02 | β| ≤ 0.27) and indirect associations via test scores (0.01 | β| ≤ 0.07). Conscientiousness explained discrepancies between teacher-assigned grades and students’ scores in domain-specific standardized tests to a greater extent than any of the other Big Five personality traits. Our findings suggest that students that are more conscientious may invest more effort to accomplish classroom goals, but fall short of mastery.

## Introduction

Student personality, especially conscientiousness, is crucial for academic achievement in secondary school (Andersen et al., 2020; Westphal et al., 2020b). Personality research has documented the necessity of examining the relationships between student personality and different indicators of student achievement (Oswald et al., 2004). While six-factor models (e.g., HEXACO, Ashton et al., 2004) and three-factor models (e.g., Paunonen et al., 2003) of personality emerged in cross-cultural research in particular, the literature on educational research has focused on the popular Big Five personality traits. As such, a number of studies has examined whether the Big Five personality traits in students differentially relate to different achievement outcomes, such as standardized test scores and teacher-assigned grades (Spengler et al., 2013; Tetzner et al., 2019). Overall, the most consistent relationships emerged between secondary-school students’ conscientiousness and their teacher-assigned grades (Poropat, 2009). Theoretical models, such as the Invest-and-Accrue Model (Hill and Jackson, 2016) “consider[s] the potential for similar mechanisms and pathways to link conscientiousness to life outcomes across multiple domains” (p. 141). However, while students’ conscientiousness is linked to their teacher-assigned grades across multiple domains, students’ conscientiousness is less consistently associated with scores in standardized achievement tests (e.g., Meyer et al., 2019).

Research on teachers’ grading practice has meanwhile established that a substantial amount of variance in teacher-assigned grades can be accounted for by students’ standardized test scores (25 to 35%; Bowers, 2011). Moreover, discrepancies between these two indicators of student achievement (i.e., teacher-assigned grades and standardized test scores) can be explained by students’ work habits (Hochweber et al., 2014; Westphal et al., 2016). In the light of these results, similar mechanisms that link conscientiousness to important life outcomes (Hill and Jackson, 2016), like high academic engagement, may be differentially relevant for different indicators of achievement. The present study therefore aims to combine both strands of research, i.e., research on teachers’ grading practice and on personality research. As such, we examine the amount of variance in teacher-assigned grades that can be explained by students’ standardized test scores, using a large and representative sample of ninth-grade students from the German National Educational Panel Study (NEPS). More importantly, we probe whether the Big Five personality traits in students, especially students’ conscientiousness, significantly help explain discrepancies between teacher-assigned grades and students’ domain-specific standardized test scores and thus why some students get more favorable grades than other students with similar test scores.

### Personality and Academic Achievement

Personality traits describe an individual’s typical patterns of cognitions, affects, and behaviors as they occur across various situations and contexts (Fleeson, 2001). The five-factor model—known as the “Big Five”—is the most established model for representing personality differences and consists of five broad domains: Openness, conscientiousness, extraversion, agreeableness, and emotional stability (McCrae and Costa, 1997; for six-factor models, e.g., HEXACO, see Ashton et al., 2004; for three-factor models, see e.g., Paunonen et al., 2003). These five dimensions have been replicated for different age groups including children and adolescents (Soto et al., 2008). These Big Five personality traits in students are highly relevant for academic achievement in secondary school and have been linked to students’ scores in standardized achievement tests (Spengler et al., 2013; Andersen et al., 2020), teacher-assigned grades (Laidra et al., 2007), and grade retention (Westphal et al., 2020b). Empirical studies identified different Big Five personality traits as important precursors for academic achievement in terms of standardized test scores (openness: e.g., Spengler et al., 2013; Waiyavutti, 2019; conscientiousness: e.g., Andersen et al., 2020; extraversion and agreeableness: e.g., Tetzner et al., 2019; emotional stability: e.g., Spengler et al., 2013) and teacher-assigned grades (openness: e.g., Steinmayr and Spinath, 2007; conscientiousness: e.g., Laidra et al., 2007; Spinath et al., 2010; Spengler et al., 2013; Rahafar et al., 2016; Zhang and Ziegler, 2016; Waiyavutti, 2019; Westphal et al., 2020a; extraversion: e.g., Steinmayr and Spinath, 2007; Spinath et al., 2010; agreeableness: e.g., Steinmayr and Spinath, 2007; emotional stability: e.g., Spinath et al., 2010). Yet, there is some evidence from meta-analytical studies that students’ conscientiousness may be of particular relevance for teacher-assigned grades in secondary school (Poropat, 2009). In contrast, empirical results are more heterogeneous and the picture is less clear for standardized test scores.

### Teacher-Assigned Grades and Standardized Test Scores as Indicators of Different Achievement Aspects

The question of the extent to which teacher-assigned grades actually measure student achievement has stimulated educational research for many years (Borghans et al., 2016). Professional guides encourage teachers to rely primarily on students’ achievement of the relevant learning goals in class when assigning final report card grades (e.g., Brookhart, 2004; Linn and Miller, 2005; Brookhart et al., 2016). Thus, Brookhart (2004) underlines that, first and foremost, grades should function as a reliable signifier of student achievement in class for the students themselves and their parents. McMillan (2001) surveyed more than 1,400 secondary-school teachers who reported that they take, to some extent, students’ work habits, effort, and participation in class into account when assigning grades. Empirical research comparing teacher-assigned grades to students’ scores in large-scale standardized tests, which measured domain-specific achievement (e.g., Bowers, 2011; Hochweber et al., 2014; Westphal et al., 2016), indicates that students’ scores in standardized achievement tests explain 25 to 35% of the variance in teacher-assigned grades (Bowers, 2011; although some studies report lower associations, e.g., Westphal et al., 2020a). Willingham et al. (2002) showed that discrepancies between teacher-assigned grades and standardized test scores can be attributed to the observable behavior of students, such as participation in class and homework completion. Other studies similarly found that student effort or motivation explain, to a substantial degree, why students achieve better or worse teacher-assigned grades than their scores in standardized tests would suggest (Hochweber et al., 2014; Westphal et al., 2016). Thus, teacher-assigned grades and standardized test scores measure slightly different aspects of achievement and achievement-related behavior, respectively.

### The Process by Which Students’ Personality Traits Might Affect Teacher-Assigned Grades and Test Scores

Several potential pathways linking personality to student achievement have been described (Poropat, 2014). Conscientiousness has been linked to effortful control (De Pauw et al., 2009) and self-control (MacCann et al., 2009), both of which could result in more time spent learning (Poropat, 2014). This is in line with the Invest-and-Accrue Model stating that highly conscientious individuals would be “devoting current resources (energy, time, assets) with the intent of achieving current or future success in an important life domain” (Hill and Jackson, 2016, p. 142). Experiencing that their striving indeed pays off will—in the sense of a feedback loop—strengthen their conscientiousness even more. Gaining benefits will subsequently enable them to invest energy in multiple domains. Beyond conscientiousness, openness is also seen as an investment trait, but in the sense that it is characterized by “stable individual differences in the tendency to seek out, engage in, enjoy, and continuously pursue opportunities for effortful cognitive activity” (von Stumm et al., 2011, p. 225). Thus, more open students may typically mobilize more cognitive effort which is in line with findings showing that openness may improve achievement in school because open students may be better able to utilize learning strategies (Blickle, 1996), may be better critical thinkers (Bidjerano and Dai, 2007), and may have higher achievement motivation (Tempelaar et al., 2007). More emotionally stable students, it has been suggested, may be less easily distracted from learning tasks, leading in turn to more time spent on these tasks and subsequently higher levels of achievement (De Raad and Schouwenburg, 1996).

The Invest-and-Accrue Model could be utilized to explain why students’ conscientiousness is consistently associated with teacher-assigned grades, but less consistently so with standardized test scores. As such, students that are more conscientious may invest more resources to accomplish classroom goals than less conscientious students. As a consequence, students who achieve similar scores in standardized achievement tests, but are more conscientious, may receive better grades. This is in line with the notion that teachers reward the classroom behavior of highly conscientious students by assigning those students grades that are more positive than their actual academic achievement might warrant (e.g., Spengler et al., 2013). Accordingly, De Raad and Schouwenburg (1996) highlighted that different facets of conscientiousness—i.e., attributes such as self-controlled, well-organized, and mature—have been cited as being core characteristics in studies describing profiles of what might be considered an ideal student. The question arises, then, of whether students’ with specific Big Five personality traits achieve better grades because of teachers’ grading practices or because of the relation of these characteristics to their actual achievement. Spengler et al. (2013) raised a similar question, using two large samples of secondary school students to examine whether students’ personality traits showed similar associations with teacher-assigned grades when compared to standardized test scores. Their results showed that teacher-assigned grades were mainly associated with conscientiousness, while standardized test scores were mainly related to openness (Spengler et al., 2013). The authors suggested that their findings could be attributed to the fact that “teachers evaluate not only students’ abilities but also their studiousness when making decisions about grades” (Spengler et al., 2013, p. 621).

### The Present Study

The present study expands the bounds of research on teachers’ grading by unraveling the relationship between teacher-assigned grades, student scores in standardized tests, and students’ personality traits. We examined whether discrepancies between teacher-assigned grades and students’ standardized achievement scores were systematically attributable to any of the Big Five personality traits in students. We utilized data from the National Educational Panel Study (Blossfeld et al., 2011) to explore this relationship in a representative sample of ninth-grade students in Germany, looking separately for teacher-assigned grades in mathematics and German.

As outlined in the introduction, on the one hand, there is a broad research literature showing that students’ scores in standardized achievement tests explain a substantial amount of variance in teacher-assigned grades (e.g., Bowers, 2011). Discrepancies between teacher-assigned grades and scores in domain-specific standardized tests, on the other hand, can be partially attributed to students’ work habits (e.g., Willingham et al., 2002; Hochweber et al., 2014). The higher investment in classroom goals that highly conscientious students show—as we would expect based on the Invest-Accrue-Model—could help explain why these students receive better teacher-assigned grades despite similar scores in standardized achievement tests. One method that may be useful in disentangling the role of personality in students’ levels of ability and studiousness is the analysis of indirect effects. Given these findings, we formulated the following hypotheses.

Hypothesis 1: We expected that students’ scores in standardized achievement tests in mathematics and German would explain a substantial amount of variance in teacher-assigned grades in mathematics and German.

Hypothesis 2: We hypothesized that the Big Five personality traits in students—especially conscientiousness—would explain teacher-assigned grades in mathematics and German over and above students’ scores in standardized achievement tests in mathematics and German.

Hypothesis 3: We expected that the association between students’ Big Five personality traits and teacher-assigned grades would be at least partially explained by students’ scores in standardized achievement tests.

## Materials and Methods

### Sample

We employed data from the National Educational Panel Study (NEPS; Blossfeld et al., 2011), a longitudinal multi-cohort study, administered in all 16 German federal states, and which focused on educational processes and competence development. The NEPS is carried out under the supervision of the German Federal Commissioner for Data Protection and Freedom of Information (BfDI), in coordination with the German Standing Conference of the Ministers of Education and Cultural Affairs (KMK), and the Educational Ministries of the respective federal states. Other published manuscripts have used data from the NEPS to examine teacher-assigned grades (Bayer and Zinn, 2018; Brandt et al., 2020). Bayer and Zinn (2018) have used data from the NEPS to test the relationship between teacher-assigned grades and students’ standardized test scores without incorporating students’ Big Five personality traits. Brandt et al. (2020) examined the associations between Big Five personality traits and teacher-assigned grades based on the NEPS, but they did not take standardized test scores in reading and mathematics into account when predicting teacher-assigned grades. The data protection unit of the Leibniz Institute for Educational Trajectories (LIfBi) has approved all instruments and data collection procedures. Participation in the NEPS was voluntary. The parents of all participants gave informed consent for their children to participate in the study, which they are able to revoke at any time. The sample comprised *N* = 16,425 ninth-grade students assessed in the first and second wave, i.e., at the beginning and at the end of the 2010–11 school year. For our study, we only included students in general secondary schools (but not remedial schools), who were surveyed along with at least five of their classmates (in other words, we excluded classes with class sizes of five or less students). Our final sample therefore consisted of *N* = 14,710 students in 922 classes. Fifty percent of students were female. The mean age of students was 14.7 years (*SD* = 0.7). On average, there were 16 students per class.

### Measures

In our analyses, we incorporated teacher-assigned grades, scores in standardized achievement tests in mathematics and German as well as indicators of students’ Big Five personality characteristics.

#### Teacher-Assigned Grades

Students reported their mid-year grades obtained in grade nine in mathematics and German (Wave 2). In the German school system, students receive numeric grades from 1 to 6, where a grade of 1 marks excellent achievement, and a grade of 6 reflects unsatisfactory achievement. For our analyses, we reverse-coded the grades so that positive regression coefficients reflect positive relationships. Thus, a grade of 6 denoted excellent achievement, while a grade of 1 reflected unsatisfactory achievement.

#### Scores in Standardized Achievement Tests

Standardized test scores for mathematics (Wave 1) and reading (Wave 2) were available. To obtain standardized test scores for mathematics, the NEPS applied 22 items from four different content areas (quantity; space and shape; change and relationships; data and chance) and six different cognitive processes (arguing; communicating; modeling; problem-solving; representing; applying technical skills) with content areas distributed across the items (Durchhardt and Gerdes, 2013). The items had a multiple-choice response format (and a short constructed response in the case of one item). Although different content areas were assessed, a unidimensional partial credit model was found to fit the data well (Durchhardt and Gerdes, 2013). WLE reliability was high (0.79; Durchhardt and Gerdes, 2013).

The NEPS measured students’ reading competence using 33 items that assessed three cognitive requirements (finding information in text; drawing text-related conclusions; reflecting and assessing) and different text functions (informational texts; instructional texts; advertising texts; commentaries or arguments; literary texts; Haberkorn et al., 2012). A partial credit model fitted the data well and WLE reliability was high (0.75; Haberkorn et al., 2012).

#### Personality Characteristics

Students’ personality traits were measured using the German 10-item version (BFI-10; Rammstedt and John, 2007) of the Big Five Inventory (John et al., 1991) in Wave 1. The BFI-10 assesses each of the Big Five traits using two items (one positively poled and one negatively poled) from the original scales of the Big Five Inventory. It determines to what extent a person is “outgoing, sociable” vs. “reserved” (Extraversion); “tends to be lazy” vs. “does a thorough job” (Conscientiousness); is “relaxed, handles stress well” vs. “gets nervous easily” (Emotional Stability); has “an active imagination” vs. “few artistic interests” (Openness to Experience); “is generally trusting” vs. “tends to find fault with others” (Agreeableness). Reasonable stabilities for all BFI-10 subscales have been demonstrated (test-retest reliabilities; Gosling et al., 2003). Each item was answered on a 5-point scale (1 = does not apply at all to 5 = fully applies).

### Statistical Analyses

We used Structural Equation Models (SEMs) with MPlus 7.4 (Muthén and Muthén, 2015) to test our hypotheses. We first specified an exploratory structural equation modeling approach (ESEM), which combines confirmatory (CFA) and exploratory factor analysis (EFA) in one model (Marsh et al., 2014). To account for well-known difficulties in replicating the five-factor structure we modeled students’ Big Five personality traits as EFA factors. To identify all factors, their variance was fixed to one and all loadings were estimated freely. To account for “yes-saying” (acquiescence), we specified a response style factor (Aichholzer, 2014). We used oblique geomin rotation. Each item showed the highest loading on the corresponding factor. Thus, the factor solution reflected the theoretically assumed five-factor structure. We have outlined the factor loadings and factor correlations in Supplementary Table 1. Beyond the latent Big Five dimensions, we included manifest indicators to capture teacher-assigned grades and standardized test scores. To estimate our model, we used maximum likelihood estimation. To handle the dependency that resulted from the nested data structure (with students clustered within classes), we used the option type = complex in Mplus (adjusting the standard errors for the model parameters; classroom was the level 2 unit). We used bootstrapping to estimate confidence intervals. To estimate how much variance in teacher-assigned grades can be explained by standardized test scores, we specified a model that included grades in mathematics and German as dependent variables and students’ standardized test scores in mathematics and German as independent variables (Model 1). We modeled correlations between our two dependent variables as well as correlations between the different standardized test scores. Next, we took the personality traits into account to test whether students’ Big Five personality traits would explain incremental variance in teacher-assigned grades over and above standardized test scores (Model 2). When specifying Model 2, we also estimated indirect effects of each Big Five personality trait on teacher-assigned grades via standardized test scores (using the command model indirect in Mplus). Model fit was evaluated based on well-established criteria (CFI > 0.95−0.97; RMSEA < 0.05−0.08, SRMR < 0.05−0.10; Hu and Bentler, 1998; Schermelleh-Engel et al., 2003). The average percentage of missing values was 2.51%. We used the robust maximum likelihood (MLR) estimator in conjunction with the full-information maximum-likelihood approach (FIML; Enders, 2001) to obtain appropriate estimates and standard errors.

## Results

**FIGURE 1:**
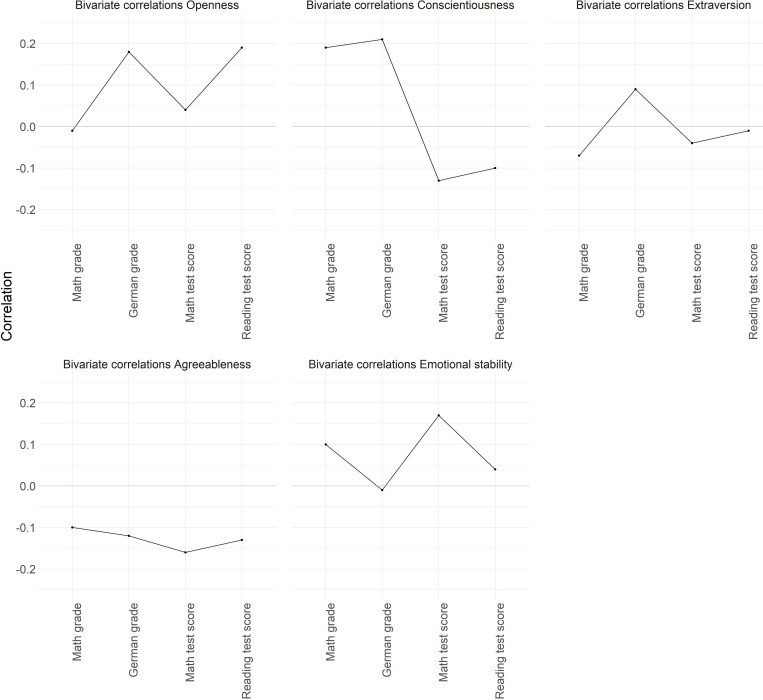
Bivariate correlations of the Big Five personality traits with teacher-assigned grades and standardized test scores (in mathematics and German).

Descriptive statistics between all study variables are presented in Table 1. In addition, bivariate correlations between the latent Big Five personality traits in students and teacher-assigned grades and standardized test scores are depicted in Figure 1. Here we can see that conscientiousness was most closely related to teacher-assigned grades in both subjects (*r* = 0.19 for mathematics to *r* = 0.21 for German). For openness and teacher-assigned grades in German, we found a correlation coefficient of *r* = 0.18, while all other correlations between teacher-assigned grades and personality traits were | r| = 0.12 or below. In addition, reading test scores showed the highest correlations with openness (*r* = 0.19), agreeableness (*r* = −0.13), and conscientiousness (*r* = −0.10). Standardized math test scores were most closely associated with emotional stability (*r* = 0.17), agreeableness (*r* = −0.16), and conscientiousness (*r* = −0.13). All other correlations between students’ standardized test scores and personality traits did not exceed | r| = 0.04.

**TABLE 1:** Descriptive statistics for BFI-10 items, teacher-assigned grades and standardized test scores.

	M	SD	Range	Skewness/Kurtosis	1	2	3	4	5	6	7	8	9	10	11	12	13
1 O Item 1	3.73	1.08	1–5	–0.49/–0.42													
2 O Item 2*	2.78	1.75	1–5	0.21/–1.09	–0.28												
3 C Item 1	3.54	0.87	1–5	–0.19/–0.36	0.08	–0.08											
4 C Item 2*	3.23	1.38	1–5	–0.16/–0.80	0.04	0.11	–0.37										
5 E Item 1	3.51	0.90	1–5	–0.22/–0.31	0.17	–0.01	0.05	0.00									
6 E Item 2*	2.63	1.24	1–5	0.13/-0.69	–0.06	0.03	0.06	0.03	–0.46								
7 A Item 1	3.38	1.07	1–5	–0.32/–0.37	0.08	–0.04	0.07	0.00	0.12	0.03							
8 A Item 2*	2.85	1.07	1–5	0.17/–0.50	0.05	0.08	–0.10	0.22	0.14	–0.14	–0.11						
9 ES Item 1	3.29	1.14	1–5	–0.15/–0.57	0.07	0.06	0.04	0.14	0.14	–0.03	0.04	0.00					
10 ES Item 2*	2.84	1.19	1–5	0.16/–0.65	–0.01	0.00	0.01	0.04	–0.22	0.31	0.08	–0.01	–0.26				
11 G math	4.02	1.05	1–6	–0.11/–0.39	–0.01	0.01	0.18	–0.09	–0.03	0.07	0.00	0.00	0.02	–0.05			
12 G German	4.13	0.70	1–6	–0.09/–0.03	0.09	–0.10	0.18	–0.12	0.09	–0.04	–0.01	0.04	–0.04	–0.04	0.39		
13 T math	0.02	1.48	–4.37–4.62	0.69/0.78	0.06	0.02	0.00	0.13	0.00	0.06	–0.01	0.10	0.07	–0.08	0.36	0.22	
14 T reading	–0.02	1.60	–4.75–3.30	0.13/0.05	0.14	0.04	–0.01	0.09	0.04	0.04	–0.03	0.08	–0.01	–0.03	0.19	0.29	0.54

O, openness; C, conscientiousness; E, extraversion; A, agreeableness; ES, emotional stability; G, grade; T, standardized test score. *Inverse item (not reverse-coded).

### Variance in Teacher-Assigned Grades Explained by Students’ Standardized Test Scores

The first aim of this study was to estimate to what extent students’ standardized test scores would explain the variance in teacher-assigned grades. We therefore specified a SEM in which teacher-assigned grades in mathematics and German were regressed on standardized test scores (Table 2, Model 1). The model showed a satisfactory fit, CFI = 0.986, RMSEA = 0.036, SRMR = 0.018. We found that students’ standardized test scores in mathematics predicted 11.9% of the variance in teacher-assigned grades in mathematics. In German, standardized scores in reading predicted 8.7% of the variance in teacher-assigned grades.

**TABLE 2:** Predicting teacher-assigned grades in mathematics and German.

	Mathematics			German		
		
	β	p	95% CI	β	p	95% CI
**Model 1**						
Test score^*a*^	0.35	0.000	[0.33, 0.36]	0.30	0.000	[0.27, 0.32]
*R*^2^		11.9%			8.7%	
CFI			0.986			
RMSEA			0.036			
SRMR			0.018			
**Model 2**						
**Direct effects**						
Test score^*a*^	0.34	0.000	[0.32, 0.36]	0.27	0.000	[0.24, 0.29]
Openness	–0.04	0.003	[−0.07, −0.01]	0.11	0.000	[0.07, 0.14]
Conscientiousness	0.27	0.000	[0.24, 0.30]	0.26	0.000	[0.23, 0.28]
Extraversion	–0.07	0.000	[−0.10, −0.05]	0.06	0.000	[0.04, 0.09]
Agreeableness	–0.12	0.000	[−0.16, −0.08]	–0.19	0.000	[−0.25, −0.13]
Emotional stability	0.08	0.000	[0.05, 0.11]	0.02	0.143	[−0.01, 0.06]
**Indirect effects**						
Openness	0.03	0.000	[0.02, 0.05]	0.06	0.000	[0.05, 0.07]
Conscientiousness	–0.03	0.000	[−0.04, −0.02]	–0.03	0.000	[−0.04, −0.02]
Extraversion	–0.03	0.000	[−0.04, −0.02]	–0.01	0.001	[−0.02, −0.01]
Agreeableness	–0.07	0.000	[−0.09, −0.05]	–0.04	0.000	[−0.06, −0.03]
Emotional stability	0.06	0.000	[0.05, 0.08]	0.02	0.001	[0.01, 0.03]
*R*^2^		19.6%			18.4%	
CFI			0.979			
RMSEA			0.034			
SRMR			0.015			

*Coefficients are standardized. ^*a*^Mathematics/reading test score.*

### Incremental Variance in Teacher-Assigned Grades Explained by Student Personality

In a second step, we additionally took the Big Five personality traits in students into account (Table 2, Model 2). The model fit was acceptable, CFI = 0.979, RMSEA = 0.034, SRMR = 0.015. Students’ personality traits additionally explained 7.7% of the variance in teacher-assigned grades in mathematics and 9.7% of the variance in teacher-assigned grades in German. When controlling for Big Five personality traits, the effect of students’ standardized test scores on teacher-assigned grades remained stable in both subjects (β = 0.34 for mathematics test scores; β = 0.27 for reading test scores). In addition, we found that students’ conscientiousness was the personality trait that best predicted teacher-assigned grades in both subjects (β = 0.27 in mathematics; β = 0.26 in German). In mathematics, all other personality traits also statistically significantly predicted teacher-assigned grades to a significant degree (β = −0.04 for openness; β = −0.07 for extraversion; β = −0.12 for agreeableness; β = 0.08 for emotional stability). Thus, students with a higher conscientiousness and emotional stability obtained better grades, while higher openness, extraversion and agreeableness were associated with lower grades. In German, openness (β = 0.11), extraversion (β = 0.06), and agreeableness (β = −0.19) were also statistically significant predictors of teacher-assigned grades. Higher openness, higher extraversion, and lower agreeableness were associated with better grades in the subject.

### Analyses of Indirect Effects of the Personality-Grade Relationships via Standardized Test Scores

To examine whether the association between students’ personality traits and teacher-assigned grades might be partially explained by students’ standardized test scores, we modeled indirect effects of personality traits on grades via test scores when specifying our SEM (Table 2, Model 2). All indirect effects were statistically significant, but standardized regression coefficients were small (| β| ≤ 0.07). We found negative indirect effects of agreeableness (β = −0.07/−0.04 in mathematics/German), conscientiousness (β = −0.03 in mathematics and German), and extraversion (β = −0.03/−0.01 in mathematics/German) and positive indirect effects of openness (β = 0.03/0.06 in mathematics/German) and emotional stability (β = 0.06/0.02 in mathematics/German) on teacher-assigned grades in mathematics and German. These results remained virtually identical when taking gender, socioeconomic status, and first-language status as covariates into account (see Supplementary Table 2).

## Discussion

Empirical research points to the importance of students’ Big Five personality traits in educational outcomes (e.g., Poropat, 2014). In secondary school, students’ conscientiousness in particular seems to be highly relevant, with teachers assigning more favorable grades to students that are more conscientious (e.g., Spengler et al., 2013). In contrast, empirical results on the role of secondary-school students’ conscientiousness for the scores students receive in domain-specific standardized tests are mixed, with a large number of studies indicating that conscientiousness is of no particular relevance to these test scores (e.g., Meyer et al., 2019). The Invest-and-Accrue Model argues that similar principles explain why conscientiousness affords benefits in multiple outcomes—i.e., conscientious individuals investing more energy, which leads to success, which in turn encourages conscientious behavior (Hill and Jackson, 2016). However, these principles may differentially affect different indicators of educational success. As such, more diligent work habits seem to explain why some students receive better grades despite similar scores in domain-specific standardized-achievement tests (e.g., Willingham et al., 2002). The present study aims to clarify whether students’ Big Five personality traits, especially conscientiousness, explain discrepancies in teacher-assigned grades and domain-specific standardized test scores.

Firstly, we found that standardized test scores explained between 8.7 and 11.9% of the variance in teacher-assigned grades, which is less than what has been found for other countries, for instance the United States (25 to 35%, Bowers, 2011). Previous research conducted in Germany similarly found that standardized test scores explain a smaller amount of variance in grades (e.g., 11.6% in a study measuring German secondary school students’ achievement in mathematics, Hochweber et al., 2014) than in other countries. In addition, standardized tests designed to capture the components from the German national educational standards for German and mathematics in secondary school explained between 20 and 28% of the variance in teacher-assigned grades in mathematics and German (Nachtigall, 2015, 2018). The standardized tests that we used for the present study correspond to the German national educational standards for mathematics and German and to the framework in the Program for International Student Assessment (PISA; Institute for Educational Quality Improvement, n.d.; Neumann et al., 2013). Thus, differences in the curricular validity of the standardized tests may be responsible for the fact that standardized test scores in our study explained a smaller amount of variance in grades than in other studies conducted in Germany (Nachtigall, 2015, 2018).

Secondly, we found that the Big Five personality traits in students explained a substantial amount of incremental variance in teacher-assigned grades beyond domain-specific standardized test scores (8% in mathematics and 10% in German). Of the Big Five personality traits, students’ conscientiousness and agreeableness were the best predictors of teacher-assigned grades. This finding is in line with the extensive body of literature showing that conscientiousness is particularly important for achieving good grades in secondary school (for meta-analytical evidence, see Poropat, 2009). Based on the Invest-Accrue-Model, this finding may reflect the fact that “conscientious individuals will perform actions that tend to maximize success” in important life domains, such as education (Hill and Jackson, 2016, p. 143). In addition, our finding that agreeableness is negatively linked to teacher-assigned grades could be explained by research showing that individuals that score high on agreeableness find competitive situations less rewarding, more challenging, and more problematic than individuals low on agreeableness (Graziano et al., 1997). On the other hand, there is an indication that expressive independence—a behavior that puts the focus on expressing one’s own ideas, instead of focusing on social responsibility and adjusting to external requirements—is associated with better teacher-assigned grades (Stephens et al., 2014). A possible explanation may therefore be that teachers perceive a more agreeable student as less confident in their own ideas than a less agreeable student with a similar level of achievement, and that teachers then interpret the student’s behavior as reflecting lower achievement. The fact that agreeableness is more closely associated with teacher-assigned grades in German than in mathematics may also help explain why findings are more heterogeneous in terms of the role of agreeableness in grading.

Thirdly, our findings indicate that the relationship between the Big Five personality traits in students and teacher-assigned grades is composed of both direct pathways and indirect pathways via standardized test scores. On the one hand, our results therefore support the rationale that students with certain personality traits achieve better scores in a more objective measure of achievement, such as standardized tests, and in turn receive better grades. Our findings (i.e., indirect association of openness and grades) are in line with the notion that students with a higher openness achieve higher scores in achievement tests and receive more favorable grades because they use learning strategies more appropriately, exhibit a higher achievement emotion, and may be better critical thinkers (Blickle, 1996; Bidjerano and Dai, 2007; Tempelaar et al., 2007). In addition, our findings are consistent with research by Graziano et al. (1997) showing that participants high on agreeableness may perceive competition as threatening, which could result in lower achievement and, as a consequence, lower grades. Our results (i.e., indirect association of emotional stability and grades) may also support the idea that more emotionally stable students are less vulnerable to being distracted and therefore spend more time on tasks (De Raad and Schouwenburg, 1996), which may in turn lead to better test scores and higher grades.

Overall, especially when considering conscientiousness, the strength of the direct association between students’ conscientiousness and teacher-assigned grades (β ≥ 0.26) was much larger than the strength of the indirect association via test scores (| β| = 0.03). Our results hold when we additionally take students’ gender, socioeconomic background, and first-language status into account (Supplementary Table 2). Thus, this finding backs up the argument that—based on the Invest-and-Accrue Model (Hill and Jackson, 2016)—highly conscientious students are more invested in classroom goals. Such an investment may not pay off in higher domain-specific achievement as would be evident in standardized achievement tests. However, it may contribute to the well-established finding that students that are more conscientious receive better grades (e.g., Laidra et al., 2007; Spinath et al., 2010; Spengler et al., 2013). In addition, our results are consistent with and add to research on teachers’ grading practices, which has repeatedly shown that observable student behavior—which is indicative of students’ effort and motivation, like classroom participation and homework completion—explains discrepancies between students’ grades and test scores (Willingham et al., 2002; see also Hochweber et al., 2014; Westphal et al., 2016). These findings could also be explained by teachers’ grading strategies being partially responsible for the relationship between teacher-assigned grades and students’ personality traits, such as conscientiousness. This may indicate that teachers value the classroom behavior of highly conscientious students more highly, which they then reward by assigning those students grades that are more positive than their actual academic achievement might warrant (for a similar suggestion see Spengler et al., 2013). Teachers themselves also reported that they took these behaviors into account favorably when assigning grades (McMillan, 2001). Indeed, in informal conversations, teachers described their higher-performing students as “caring more about doing well in school,” having “the will’ to complete their homework, pay[ing] attention in class, and otherwise work[ing] hard” (Duckworth et al., 2015, p. 14). According to Poropat, the social desirability of some Big Five personality traits “may also be important, resulting in a positive halo effect that may raise teachers’ ratings of children’s academic performance” (Poropat, 2014, p. 242). To sum up, students’ conscientiousness explains, to some extent, the discrepancies between teacher-assigned grades and students’ scores in domain-specific standardized tests.

### Limitations and Future Research

The present study has several limitations. For a start, we cannot rule out the possibility that the direct links found in our study between students’ personalities and the grades that teachers assign them might be partially attributable to higher student achievement in subdomains that were not measured by standardized achievement tests. Thus, the use of broad standardized tests adapted even more specifically to the given school’s curriculum would be a welcome addition to future research.

Secondly, we captured student personality using a short scale. Short scales sometimes exhibit problems in terms of their factorial validity. The use of short scales, which assess personality traits more narrowly, might also lead researchers to underestimate the role of personality in educational outcomes, including academic achievement (Credé et al., 2012). At the same time, Rammstedt and John (2007) found that their short form gauged 70% of the variance of the long version of the Big Five Inventory and, among different short scales, the short version of the Big Five Inventory by Rammstedt and John (2007) demonstrated the highest criterion validity (Credé et al., 2012). While our results are in line with previous studies that use long versions of personality scales (Poropat, 2014), future research should incorporate longer versions when testing the role of personality in teachers’ grading, given the potential limitations of short personality scales. In addition, future studies should shed light on the question of how different subdimensions of Big Five personality traits relate to different indicators of academic achievement.

Thirdly, our sample consisted of secondary school students in the ninth grade and we cannot generalize our findings to other age groups. There is some indication that different personality traits play a role for teacher-assigned grades in primary than in secondary school (Laidra et al., 2007) and therefore more research with primary students and other age groups is needed. To add to this, the majority of studies have been conducted in Western countries (for exceptions see e.g., Rahafar et al., 2016; Zhang and Ziegler, 2016; Waiyavutti, 2019). Future studies should therefore focus on student personality and its role for achievement in non-Western societies. As the Big Five personality traits are not always replicable in samples that are less well-educated, democratic, rich, industrialized, and non-Western (e.g., Gurven et al., 2013), researchers should complement recent findings by applying three-factor and six-factor models of personality (e.g., Paunonen et al., 2003; Ashton et al., 2004). Finally, the social relations model (e.g., Kenny et al., 2006) suggests that the relationship between the teacher (i.e., the judge) and the student (i.e., the target being judged) is a relevant aspect for interpersonal phenomena and, thus, it might be promising if future studies on teachers’ grading practice include additional measures that gauge the relationship between teacher and student.

### Practical Implications and Conclusion

In this study, we were able to disentangle the roles of the Big Five personality traits in students and standardized test scores in the grades that teachers assign them. Our findings suggest that conscientiousness explains discrepancies between teacher-assigned grades and scores in domain-specific standardized tests to a greater extent than any of the other Big Five personality traits. Although, according to the Invest-and-Accrue Model (Hill and Jackson, 2016), similar principles may link conscientiousness to important educational outcomes, these pathways may differentially affect teacher-assigned grades and standardized test scores. Our results also correspond to the idea that “grades are used to reward students for exerting effort to learn material, even if students fall short of mastery” (Kelly, 2008, p. 32). In terms of theory-development, the present study underlines the advantages of integrating empirical findings from two strands of research (personality research and research into teachers’ grading practices) when deriving conclusions about the very nature and development of student achievement in order to better understand “what’s in a grade” (Bowers, 2011, p. 141).

## Data Availability Statement

The datasets presented in this study can be found in online repositories. The names of the repository/repositories and accession number(s) can be found below: The Research Data Center at the Leibniz Institute for Educational Trajectories (RDC-LIfBi) prepares and disseminates the survey data of the German National Educational Panel Study in the form of Scientific Use Files to the scientific community. The datasets are available for download from the study website: https://www.neps-data.de/Data-Center/Data-Access/Download.

## Ethics Statement

The NEPS study is conducted under the supervision of the German Federal Commissioner for Data Protection and Freedom of Information (BfDI) and in coordination with the German Standing Conference of the Ministers of Education and Cultural Affairs (KMK) and – in the case of surveys at schools – the Educational Ministries of the respective Federal States. All data collection procedures, instruments and documents were checked by the data protection unit of the Leibniz Institute for Educational Trajectories (LIfBi). The necessary steps are taken to protect participants’ confidentiality according to national and international regulations of data security. Participation in the NEPS study is voluntary and based on the informed consent of participants. This consent to participate in the NEPS study can be revoked at any time.

## Author Contributions

AW was responsible for the planning, data analysis, and writing of this manuscript, and primary investigator on the DFG funded project. JK and MV contributed through intellectual collaboration and manuscript revisions. All authors contributed to the article and approved the submitted version.

## Conflict of Interest

The authors declare that the research was conducted in the absence of any commercial or financial relationships that could be construed as a potential conflict of interest.
