# Supplementary material for: Unraveling the Relationship Between Teacher-Assigned Grades, Student Personality, and Standardized Test Scores
Source: Front Psychol. 2021 Mar 19;12:627440. doi: 10.3389/fpsyg.2021.627440 (PMC8017135; doi:10.3389/fpsyg.2021.627440)
Supplement: Supplementary file 1 [file Table_1.DOCX]

Supplementary Material

Supplementary Table 1

Geomin Rotated Loadings of the BFI-10 Items on the Big Five Dimensions.

|  | Factor loadings | | | | |  |
| --- | --- | --- | --- | --- | --- | --- |
| I see myself as someone who … | O | C | E | A | ES |  |
| has an active imagination. (O) | .48 | -.05 | .10 | -.01 | .04 |  |
| has few artistic interests. (O-) | -.65 | -.03 | .02 | -.01 | .03 |  |
| does a thorough job. (C) | .09 | .49 | -.05 | .00 | .08 |  |
| tends to be lazy. (C-) | .02 | -.82 | -.03 | .00 | .05 |  |
| is outgoing, sociable. (E) | .03 | .01 | .68 | .12 | .03 |  |
| is reserved. (E-) | .02 | .01 | -.75 | .01 | .01 |  |
| is generally trusting. (A) | .01 | -.03 | .07 | .48 | -.09 |  |
| tends to find fault with others. (A-) | .01 | -.20 | .21 | -.31 | -.08 |  |
| is relaxed, handles stress well. (ES) | .00 | -.09 | -.03 | .07 | .60 |  |
| gets nervous easily. (ES-) | .01 | -.04 | -.27 | .06 | -.46 |  |
|  | Factor correlations | | | | |  |
|  | O | C | E | A |  |  |
| C | .12 |  |  |  |  |  |
| E | .07 | .00 |  |  |  |  |
| A | .16 | .15 | -.07 |  |  |  |
| ES | -.03 | -.02 | .21 | .07 |  |  |

*Note*. Standardized loadings from an exploratory structure equation model (ESEM) controlling for acquiescent responding. Target loadings are in boldface. O = Openness; C = Conscientiousness; E = Extraversion; A = Agreeableness; ES = Emotional stability

Supplementary Table 2

*Predicting teacher-assigned grades in mathematics and German*

|  |  | Teacher-assigned grades | | | |  |
| --- | --- | --- | --- | --- | --- | --- |
|  |  | Mathematics |  |  | German |  |
|  | *ß* | *p* | 95% CI | *ß* | *p* | 95% CI |
|  |  |  |  |  |  |  |
| *Model 2 with covariates* |  |  |  |  |  |  |
| Direct effects |  |  |  |  |  |  |

| Test score^a^ | .36 | .000 | [.34, .39] | .23 | .000 | [.21, .26] |
| --- | --- | --- | --- | --- | --- | --- |
| Openness | -.02 | .015 | [-.04, -.00] | .04 | .004 | [.01, .07] |
| Conscientiousness | .26 | .000 | [.23, .28] | .23 | .000 | [.20, .26] |
| Extraversion | -.08 | .000 | [-.10, -.05] | .06 | .000 | [.04, .09] |
| Agreeableness | -.08 | .000 | [-.12, -.04] | -.15 | .000 | [-.21, -.10] |
| Emotional stability | .07 | .000 | [.04, .10] | .05 | .006 | [.01, .08] |
| Male gender^b^ | .02 | .087 | [-.00, .05] | -.14 | .000 | [-.17, -.11] |
| Language minority^c^ | -.01 | .447 | [-.03, .01] | -.05 | .000 | [-.07, -.03] |
| HISEI | .01 | .436 | [-.01, .03] | .09 | .000 | [.06, .11] |

| Indirect effects |  |  |  | | |  |  |  |
| --- | --- | --- | --- | --- | --- | --- | --- | --- |
| Openness | .01 | .090 | [-.00, .02] | | | .02 | .000 | [.01, .03] |
| Conscientiousness | -.02 | .001 | [-.03, -.01] | | | -.02 | .000 | [-.03, -.01] |
| Extraversion | -.03 | .000 | [-.04, -.02] | | | -.01 | .000 | [-.02, -.01] |
| Agreeableness | -.04 | .000 | [-.05, -.02] | | | -.03 | .000 | [-.04, -.02] |
| Emotional stability | .04 | .000 | [.03, .06] | | | .02 | .000 | [.01, .03] |
| Male gender^b^ | .04 | .000 | [.03, .05] | | | -.04 | .000 | [-.05, -.03] |
| Language minority^c^ | -.04 | .000 | [-.05, -.04] | | | -.04 | .000 | [-.04, -.03] |
| HISEI | .10 | .000 | [.09, .12] | | | .06 | .000 | [.05, .07] |
| R² |  | 20.0% |  | | |  | 19.7% |  |
| CFI |  | .975 |  | |  |  | .964 |  |
| RMSEA |  | .032 |  |  | |  | .038 |  |
| SRMR |  | .014 |  |  | |  | .015 |  |

*Note.* Coefficients are standardized. ^a^ Mathematics/ reading test score. ^b^ Reference: female. ^c^ Reference: German is first language.

Supplementary Table 3

*Predicting standardized test scores for mathematics and reading*

|  |  | Standardized test scores | | | |  |
| --- | --- | --- | --- | --- | --- | --- |
|  |  | Mathematics |  |  | German |  |
|  | *ß* | *p* | 95% CI | *ß* | *p* | 95% CI |
| *Model 1* |  |  |  |  |  |  |
| Teacher-assigned grade | .32 | .000 | [.30, .34] | .25 | .000 | [.23, .27] |
| R² |  | 10.3% |  |  | 6.2% |  |
| CFI |  |  | .981 |  |  |  |
| RMSEA |  |  | .067 |  |  |  |
| SRMR |  |  | .040 |  |  |  |
|  |  |  |  |  |  |  |
| *Model 2* |  |  |  |  |  |  |
| Direct effects |  |  |  |  |  |  |
| Teacher-assigned grade | .22 | .000 | [.32, .36] | .16 | .000 | [.13, .19] |
| Openness | .16 | .000 | [.10, .21] | .26 | .000 | [.21, .32] |
| Conscientiousness | -.07 | .037 | [-.13, -.00] | -.05 | .052 | [-.11, .00] |
| Extraversion | -.10 | .000 | [-.14, -.05] | -.08 | .000 | [-.12, -.04] |
| Agreeableness | -.40 | .000 | [-.49, -.32] | -.38 | .000 | [-.44, -.31] |
| Emotional stability | .18 | .000 | [.12, .24] | .08 | .006 | [.02, .14] |
| Indirect effects |  |  |  |  |  |  |
| Openness | .01 | .059 | [-.00, .03] | .04 | .000 | [.03, .05] |
| Conscientiousness | .07 | .000 | [.06, .09] | .05 | .000 | [.04, .06] |
| Extraversion | -.03 | .000 | [-.04, -.02] | .01 | .164 | [-.00, .01] |
| Agreeableness | -.10 | .000 | [-.11, -.08] | -.07 | .000 | [-.09, -.06] |
| Emotional stability | .04 | .000 | [.02, .05] | .01 | .095 | [-.00, .02] |
| R² |  | 31.2% |  |  | 24.1% |  |
| CFI |  |  | .976 |  |  |  |
| RMSEA |  |  | .036 |  |  |  |
| SRMR |  |  | .014 |  |  |  |

*Note.* Coefficients are standardized.
